# 

**Published:** 1884-08

**Authors:** 


CONTENTS.

| | Page. |
|---|---|
| Cholera | 139 |
| The Sick-Room | 142 |
| Care of the Teeth | 143 |
| A Study of Leprosy | 145 |
| The Paraguay Tea Tree | 146 |
| Yellow Fever | 147 |
| Fever from Bad Milk | 147 |
| Chicago Beef | 147 |
| Vacation Visits | 149 |
| Effects of Thin Atmosphere | 149 |
| Health Alphabet | 150 |
| Kefir | 151 |
| A Lady Quelled Them | 152 |
| India-Rubber in Brazil | 153 |
| Ammonia, or Hartshorn | 154 |
| Miscellaneous | 155-56-57-58 |

